# Diethylstilbestrol mediates vascular endothelial inflammatory injury in vitro and in vivo

**DOI:** 10.1002/ctm2.47

**Published:** 2020-06-05

**Authors:** Min Zhang, Jun Shi

**Affiliations:** ^1^ Division of Cardiology TongRen Hospital Shanghai Jiao Tong University School of Medicine Shanghai China; ^2^ Shanghai Institute of Pollution Control and Ecological Security Key Laboratory of Yangtze River Water Environment Ministry of Education College of Environmental Science and Engineering Tongji University Shanghai China

Dear Editor,

Diethylstilbestrol (DES) is a common environmental contaminant that may cause adverse human health effects via endocrine disruption. Recently, DES has been implicated in the impairment of the cardiovascular system.^1^ Accumulating data from human and animal studies indicate that DES exposure contributes to carcinogenesis, reproductive system injury, and impairment of the immune system.^2^, ^3^ Nevertheless, the effects of DES on the circulatory system remains largely unknown. In this study, evidence for adverse impacts of DES in vascular ECs, both in vitro and in vivo, is presented that highlights the need for additional research focused on DES toxicity and underlying molecular mechanism. Human umbilical vein endothelial cells (HUVECs), chick embryo chorioallantoic membrane (CAM), and matrigel plug were treated with VEGF and DES. We found that DES prevented VEGF mediated action on viability, motility, and capillary network formation of HUVECs by CCK‐8, migration, and capillary tubule formation assay, respectively. Also, DES prevented VEGF‐mediated angiogenesis and vasculogenesis by CAM and subcutaneous matrigel. DES facilitated anti‐angiogenic effects on HUVECs by inducing inflammatory cytokines via YAP/Mst1–FOXO3A signaling. This study demonstrates for the first time that DES promotes a proinflammatory response in vascular endothelial cells (ECs) by inducing inflammatory cytokines both in vitro and in vivo. Exposure to DES might lead to cellular inflammation events and ECs injury, and consequently, cause vascular dysfunction increased risk of cardiovascular disease.

DES significantly suppress the growth of testis cells, stem cells, NK cell, and others. However, the effect of DES on EC angiogenesis role is still unknown. In the present study, HUVECs viability reduced blocked by treatment with DES. DES exposure significantly reduced HUVECs viability at concentrations of 25 μM or more (Figure 1A). DES also repressed viability in a time dependent manner (Figure 1B).

**FIGURE 1:**
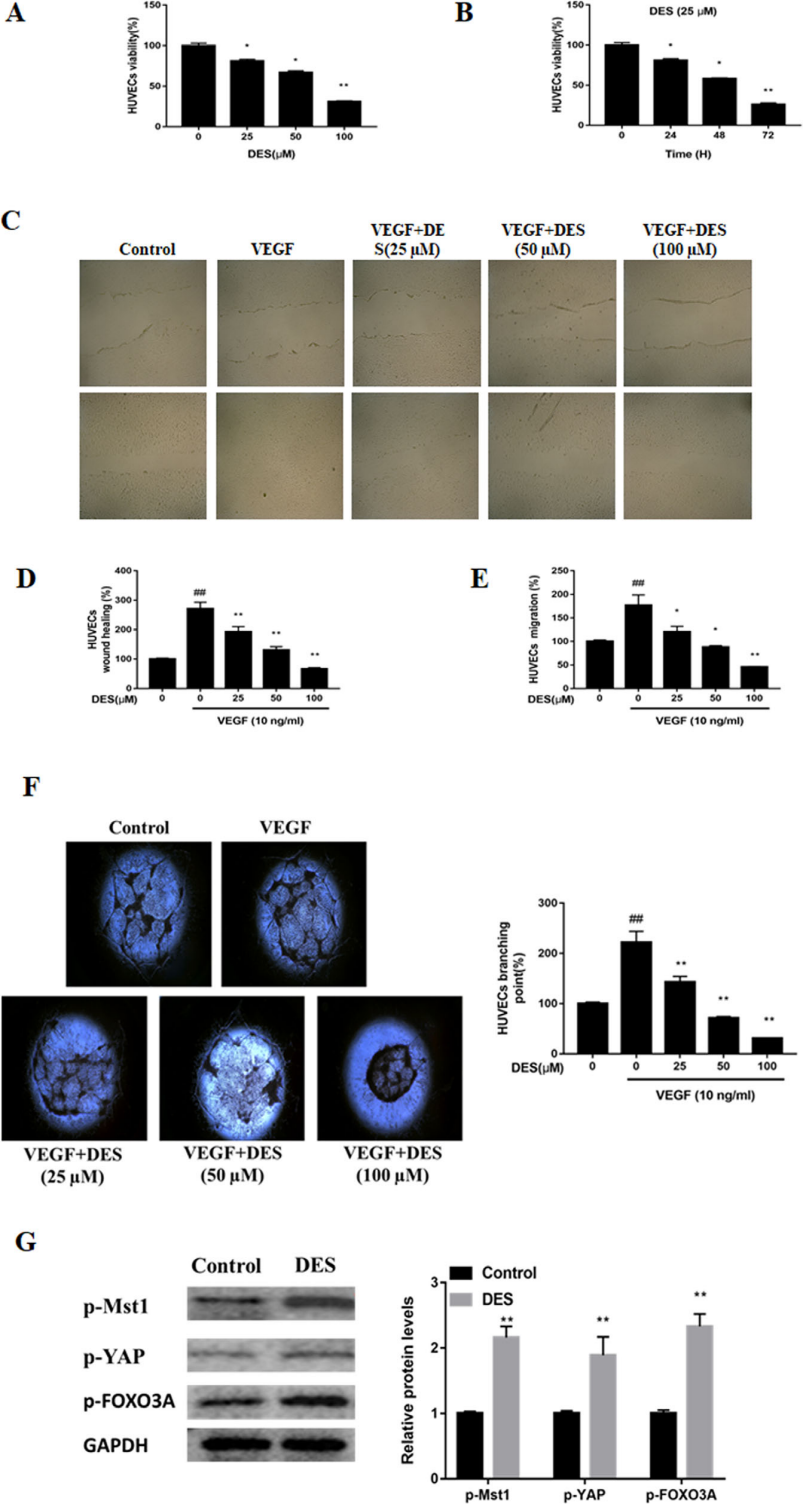
A, DES reduces the viability of HUVECs. HUVECs were treated with the 0‐100 μM of DES. After indicated times, viability was assessed using CCK‐8 assays. B, Time‐course study of viability. ^*^ *P* < .05, ^**^ *P* < .001; DES prevents VEGF‐induced chemotactic migration in HUVECs. C,D, HUVECs were exposed to10 ng/mL of VEGF and 0‐100 μM of DES; representative images of cells from in vitro wound healing assays. E, HUVECs were treated with 10 ng/mL of VEGF and 0‐100 μM of DES for 24 h, the number of cells in in vitro transwell assays. ^*^ *P* < .05, ^**^ *P* < .001; F, VEGF induces capillary‐like lumen formation in HUVECs. HUVECs were treated with 10 ng/mL of VEGF and 0‐100 μM of DES. Lumen formation of HUVECs was stopped and measured by counting lumen formations. ^#^ *P* < .05, ^##^ *P* < .001; G, Effect of DES on the activation of YAP/Mst1–FOXO3A in HUVECs. Total cell lysates were used to evaluate the levels of proteins. ^*^ *P* < .05, ^**^ *P* < .001

DES also effectively prevented chemotactic migration and capillary‐like tube formation. ECs migration contributes to angiogenesis. Wound sizes in HUVEC cultures were substantially narrowed by incubation in the presence of VEGF (10 ng/mL) for 24 h (Figure 1C,D). DES reversed VEGF‐mediated wound closure of HUVECs between edges of the scratch. Moreover, at a concentration of 100 μM, the wound area of ECs was almost reversed, from 271.76 ± 22.29% of control (VEGF treatment) to 67.21 ± 4.31 % (with DES treatment) (Figure 1C,D). Transwell assays also revealed that ECs migration rate was suppressed in by DES treatment (Figure 1E). Formation of capillary tubes was assessed after incubation for 12 h in the presence of VEGF and DES. VEGF (10 ng/mL) significantly stimulated capillary lumen formation to 225 ± 22% of the control cells (Figure 1F). DES at concentrations of 25, 50, or 100 μM dramatically diminished network formation (Figure 1F). DES induces an inflammatory response ECs, and current findings indicate that this response is due induction of vascular inflammation via inflammatory mediators and proinflammatory cytokines.

Both CAM and matrigel plug assays are frequently used for assessing angiogenesis. These methods are low‐cost, simple, and highly reproducible^4^, ^5^, ^6^ In this study, CAM assay showed that DES significantly inhibits VEGF‐mediated vessel formation (Figure 2A,B). Using Matrigel plugs, DES also notably suppressed VEGF mediated microvessel integrity. Compared to the control mice, the capillary density was significantly higher in the VEGF treated plugs, and DES reversed these effects (Figure 2C,D). VEGF‐induced more than a fourfold increase in hemoglobin levels compared to the control mice, and this increase was absent in DES‐treated plugs (Figure 2E). These data suggest that DES might inhibit angiogenesis in vivo.

**FIGURE 2:**
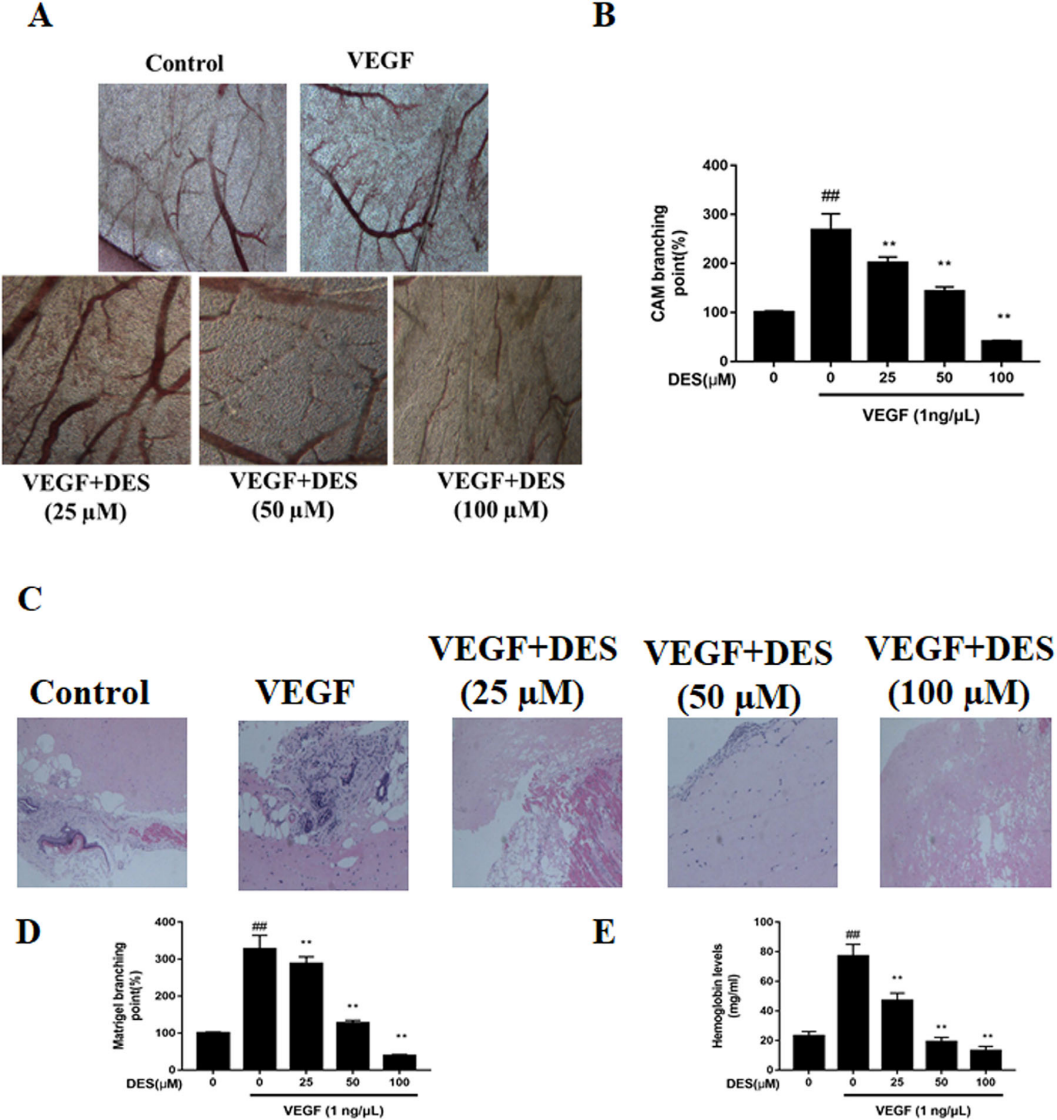
DES limited VEGF‐induced number of capillarity formation in the CAM model. A,B, The fertilized eggs were treated with 0‐100 μM of DES and 1 ng/μL of VEGF for 7 days. The left panel shows magnified blood vessels. The right panel shows relative blood vessel density in the CAM model. ^*^ *P* < .05, ^**^ *P* < .001, ^##^ *P* < .01; C,D, DES limited VEGF‐induced angiogenesis in vivo. C57BL/6 mice were injected with 0.8 mL of matrigel containing 0‐100 μM of DES and 1 ng/μL of VEGF. Matrigel plugs were obtained from mice and stained with H&E assay. E, Hemoglobin levels of the Matrigel plugs were estimated with a Drabkin's reagent kit. ^*^ *P* < .05, ^**^ *P* < .001, ^##^ *P* < .01

Increasing evidence demonstrates that FOXO3A expression inhibits cell proliferation, migration, and tube formation. FOXO3A is a key downstream target of the Hippo–YAP pathway.^7^ As an essential factor of the Hippo/YAP pathway, Mst1 is functionally upstream of FOXO3A. Mst1 elevates phosphorylation expression of FOXO3A and triggers expression of downstream genes.^8^ Activation of the Hippo–YAP pathway can induce FOXO3A, and subsequently affect EC function. Phosphorylation of Mst1, YAP, and FOXO3A were higher in the DES‐treated cells (Figure 1G), suggesting that DES exerts its impairment effect on ECs via inducing the YAP–FOXO3A signaling pathway.

In conclusion, DES impaired angiogenic function in HUVECs, including overall viability, migration, and capillary‐like lumen formation. Additionally, the anti‐angiogenic activity of DES was found both in CAM and matrigel plug in vivo assays. Impaired angiogenic function caused by DES is associated with the upregulation of YAP/Mst1–FOXO3A signaling, providing new insight into DES cytotoxicity.

## CONFLICT OF INTEREST

The authors have declared no conflict of interest.

## Supporting information

Supporting Information

## Data Availability

Data are available upon reasonable request.

## References

[1] Li YF , Canario AVM , Power DM , Campinho MA . Ioxynil and diethylstilbestrol disrupt vascular and heart development in zebrafish. Environ Int. 2019;124:511‐520. 30685453 10.1016/j.envint.2019.01.009

[2] Kioumourtzoglou MA , Coull BA , O'Reilly EJ , Ascherio A , Weisskopf MG . Association of exposure to diethylstilbestrol during pregnancy with multigenerational neurodevelopmental deficits. JAMA Pediatr. 2018;172(7):670‐677. 29799929 10.1001/jamapediatrics.2018.0727 PMC6137513

[3] Troisi R , Hatch EE , Palmer JR , et al. Estrogen metabolism in postmenopausal women exposed in utero to diethylstilbestrol. Cancer Epidemiol Biomarkers Prev. 2018;27(10):1208‐1213. 30049842 10.1158/1055-9965.EPI-18-0135 PMC6170707

[4] Xu S , Guo R , Li PZ , et al. Dexamethasone interferes with osteoblasts formation during osteogenesis through altering IGF‐1‐mediated angiogenesis. J Cell Physiol. 2019. 10.1002/jcp.28157 30671960

[5] Srivastava S , Zahra FT , Gupta N , Tullar PE , Srivastava SK , Mikelis CM . Low dose of penfluridol inhibits VEGF‐induced angiogenesis. Int J Mol Sci. 2020;21(3). 10.3390/ijms21030755 PMC7036977 31979394

[6] Zhang M , Xu Y , Jiang L . Irisin attenuates oxidized low‐density lipoprotein impaired angiogenesis through AKT/mTOR/S6K1/Nrf2 pathway. J Cell Physiol. 2019;234(10):18951‐18962. 30942905 10.1002/jcp.28535

[7] Wei B , Dui W , Liu D , Xing Y , Yuan Z , Ji G . MST1, a key player, in enhancing fast skeletal muscle atrophy. BMC Biol. 2013;11:12. 23374633 10.1186/1741-7007-11-12 PMC3606410

[8] Wang Y , Li J , Gao Y , et al. Hippo kinases regulate cell junctions to inhibit tumor metastasis in response to oxidative stress. Redox Biol. 2019;26:101233. 31212215 10.1016/j.redox.2019.101233 PMC6582208

