# Supplementary material for: Diethylstilbestrol mediates vascular endothelial inflammatory injury in vitro and in vivo
Source: Clin Transl Med. 2020 Jun 5;10(2):e47. doi: 10.1002/ctm2.47 (PMC7403659; doi:10.1002/ctm2.47)
Supplement: Supplementary file 1 — Supporting Information [file CTM2-10-e47-s001.docx]

Dear Editor,

Preexisting genetic mutations in cells may influence phe-

notypes together with other molecular biology manipula-

tions. For instance, the bladder cancer cell RT4 harbors a

TACC3-FGFR3 fusion rather than TP53 mutation (Figure 1F),

whereas the other bladder cancer cell T24 have TP53 mutation

but not FGFR3 mutation.

1

We questioned whether those pre-

existing genotypes could influence research conclusions and

systematically overviewed urothelial carcinoma-related 1589

articles either used or not used RT4 (Table S1).

We found a significant association between target gene cor-

responding pathways and RT4 usage frequency. For exam-

ple, the research was less likely to include RT4 (Figures 1A

and 1B and Table S2) if the target gene was associate with

TP53 pathways. We calculated the frequency of using RT4

and/or T24 for research on particular target gene (Table S3).

Using PPI networks,

2

we calculated the “interaction distance”

between these genes to TP53 and FGFR3. RT4 usage fre-

quency was increased in research of genes with closer rela-

tionship to FGFR3 compared to TP53 (Figure 1C). Those

results suggested the existence of latent bias in research,

that is, researcher may implicitly select consistent results

by selecting carcinoma cell lines. The preexisting carcinoma

cell line mutation landscape could determine its response

to particular molecular biology manipulation, which can be

inconsistent, even though only consistent results would be

reported.

Moreover, to define the genotype of carcinoma, cell line is

critical to the research result. State-of-the-art practices require

genotyping cell lines using short tandem repeat (STR).

3

However, STR only identifies whether cell lines are cross-

contaminated, but does not fully report the genomic changes

of cell lines. We collected eight most widely used bladder

cancer cell lines and confirmed the identity by STR. Whole-

exome sequencing (WES) of the eight cell lines together with

two conditionally reprogrammed cells (CRCs)

4

and two blad-

der cancer tissues (Table S4) showed the CRCs have good

consistency with tumor tissues; however, the cell lines showed

Dear Editor,

Preexisting genetic mutations in cells may influence phe-

notypes together with other molecular biology manipula-

tions. For instance, the bladder cancer cell RT4 harbors a

TACC3-FGFR3 fusion rather than TP53 mutation (Figure 1F),

whereas the other bladder cancer cell T24 have TP53 mutation

but not FGFR3 mutation.

1

We questioned whether those pre-

existing genotypes could influence research conclusions and

systematically overviewed urothelial carcinoma-related 1589

articles either used or not used RT4 (Table S1).

We found a significant association between target gene cor-

responding pathways and RT4 usage frequency. For exam-

ple, the research was less likely to include RT4 (Figures 1A

and 1B and Table S2) if the target gene was associate with

TP53 pathways. We calculated the frequency of using RT4

and/or T24 for research on particular target gene (Table S3).

Using PPI networks,

2

we calculated the “interaction distance”

between these genes to TP53 and FGFR3. RT4 usage fre-

quency was increased in research of genes with closer rela-

tionship to FGFR3 compared to TP53 (Figure 1C). Those

results suggested the existence of latent bias in research,

that is, researcher may implicitly select consistent results

by selecting carcinoma cell lines. The preexisting carcinoma

cell line mutation landscape could determine its response

to particular molecular biology manipulation, which can be

inconsistent, even though only consistent results would be

reported.

Moreover, to define the genotype of carcinoma, cell line is

critical to the research result. State-of-the-art practices require

genotyping cell lines using short tandem repeat (STR).

3

However, STR only identifies whether cell lines are cross-

contaminated, but does not fully report the genomic changes

of cell lines. We collected eight most widely used bladder

cancer cell lines and confirmed the identity by STR. Whole-

exome sequencing (WES) of the eight cell lines together with

two conditionally reprogrammed cells (CRCs)

4

and two blad-

der cancer tissues (Table S4) showed the CRCs have good

consistency with tumor tissues; however, the cell lines showed

**Supplementary information**

**Supplementary material and methods**

**Cell culture and treatments**

**In vitro cell viability assay**

**In vitro scratch assay**

**In vitro Transwell assay**

**In vitro lumen formation assay**

**Western blot analysis**

**Enzyme-linked immunosorbent assay**

**Chick embryo chorioallantoic membrane assay**

**Angiogenesis evaluation in vivo**

**Statistical analysis**

**Supplementary Figures**

**Figure S1**

**Supplementary material and methods**

**Cell culture and treatments**

HUVECs were obtained from Allcells (Shanghai China) and seeded in EC growth medium with an ECGM-2-MV bullet kit. Cells were cultured in a humidified incubator at 37 °C under 5% CO_2_ unless otherwise noted.

***In vitro* cell viability assay**

HUVECs (1×10^4^ cells/well) were cultured in the presence of VEGF (10 ng/ml) plus DES (Sigma-Aldrich, 0–100 µM) for 24–72 h. HUVEC viability was determined using a Cell Counting Kit-8.

***In vitro* scratch assay**

Scratched lesions were constructed, and fresh DMEM medium plus 0.5% FBS containing VEGF (10 ng/ml) alone or in combination with DES (0–100 µM, Sigma-Aldrich) were added and cells incubated for 24 h.

***In vitro* Transwell assay**

HUVECs were treated with DES (Sigma-Aldrich, 0–100 µM) for 24 h. The down insert was added with 600 µl DMEM medium containing VEGF (10 ng/ml). HUVECs (2×10^4^ cells/well) were seeded on the top insert for and incubated for 12 h. Migration indices were evaluated as previously described[21].

***In vitro* lumen formation assay**

Matrigel Matrix (Corning-Costar, 75µL) was incubated in 96 well plates for 60 min. After treatment with DES (0–100 µM) for 24 h. HUVECs (1×10^5^ cells) were seeded into 96 well plates containing VEGF (10 ng/ml) and incubated for 12 h. Lumen formation was evaluated with an Angiogenesis Analyzer plug in on ImageJ.

**Western blot analysis**

HUVECs were applied to cold RIPA Lysis buffer to obtain protein. Total proteins were transferred onto polyvinylidene difluoride membranes and blocked in skim milk for 1 h. Membranes were then incubated with primary antibodies at 4 °C overnight, and treated with HRP linked antibody anti-rabbit IgG, Specific signals were detected with an enhanced chemiluminescence system. Quantification used the image-pro plus 6.0 client, standardized by GAPDH.

**Enzyme-linked immunosorbent assay**

Inflammatory cytokines in HUVEC supernatants, including TNF-α, IL-6, and IL-1β, were detected using a commercially available ELISA kit following the manufacturer's command. The absorbance of HUVEC was measured at 450 nm using a spectrophotometer.

**Chick embryo chorioallantoic membrane assay**

In brief, fertilized eggs were placed in an incubator for 48 h and some of the shell membrane was removed. VEGF (1 ng/μL) and DES (0–100 µM) were carefully administrated onto the CAM. Chick embryos were incubated at 37 °C for another week. The vascular arrangement of the CAM was photographed under a microscope.

**Angiogenesis evaluation in vivo**

Matrigels (800 µL) mixtures containing VEGF (1 ng/μL) and DES (0–100 µM) were injected subcutaneously into the ventral area of C57BL/6 mice. After 8 days, all of mice (n=6) were euthanized, and matrigel plugs recovered. To measure neovascularization in plugs, concentrations of hemoglobin were estimated using a Drabkin reagent kit. Harvested plugs were fixed with 4% formalin, and sections incubated with hematoxylin and eosin (H&E) following manufacturer's instructions. Sections were imaged with an Olympus microscope.

**Statistical analysis**

All of results were expressed as mean ± SEM for at least three independent experiments and` analyzed using GraphPad Prism 5.0. Statistical significance was analyzed using one-way ANOVA followed by Dunnett's test or Tukey's test. *p* values less than 0.05 were considered significant.

**Supplementary Figures**

**Figure S1**

Fig. S1 Effect of DES on proinflammatory cytokines in HUVECs. The expression level of IL-6, TNF-α and IL-1β were determined by ELISA. * P<0.05, ** P<0.001
